# Blockage of autophagy pathway enhances *Salmonella* tumor-targeting

**DOI:** 10.18632/oncotarget.8251

**Published:** 2016-03-22

**Authors:** Binghong Liu, Yanan Jiang, Tiangeng Dong, Ming Zhao, Jianfu Wu, Lihui Li, Yiwei Chu, Shangyang She, Hu Zhao, Robert M. Hoffman, Lijun Jia

**Affiliations:** ^1^ Cancer Institute, Fudan University Shanghai Cancer Center, Collaborative Innovation Center of Cancer Medicine, Department of Oncology, Shanghai Medical College, Fudan University, Shanghai 200032, China; ^2^ Department of Immunology, Shanghai Medical College, Fudan University, Shanghai 200032, China; ^3^ Department of Clinical Laboratory, Children's Hospital, Maternal and Child Health Hospital of Guangxi Zhuang Autonomous Region, Nanning 530003, China; ^4^ Department of General Surgery, Zhongshan Hospital, Fudan University, Shanghai 200032, China; ^5^ Department of Surgery, University of California, San Diego, California 92103, USA; ^6^ Department of Clinical Laboratory, Huadong Hospital, Shanghai Key Laboratory of Clinical Geriatric Medicine, Research Center on Aging and Medicine, Fudan University, Shanghai 200040, China; ^7^ AntiCancer, Inc., San Diego, California 92111, USA

**Keywords:** autophagy, Salmonella typhimurium A1-R, bacteria, cancer, apoptosis

## Abstract

Previous studies have shown that strains of *Salmonella typhimurium* specifically target tumors in mouse models of cancer. In this study, we report that tumor-targeting *Salmonella typhimurium* A1-R (A1-R) or VNP20009 induced autophagy in human cancer cells, which serves as a defense response. Functionally, by knockdown of essential autophagy genes Atg5 or Beclin1 in bacteria-infected cancer cells, the autophagy pathway was blocked, which led to a significant increase of intracellular bacteria multiplication in cancer cells. Genetic inactivation of the autophagy pathway enhanced A1-R or VNP20009-mediated cancer cell killing by increasing apoptotic activity. We also demonstrate that the combination of pharmacological autophagy inhibitors chloroquine (CQ) or bafilomycin A1 (Baf A1) with tumor-targeting A1-R or VNP20009 significantly enhanced cancer-cell killing compared with *Salmonella* infection alone. These findings provide a proof-of-concept of combining autophagy inhibitors and tumor-targeting *Salmonella* to enhance cancer-cell killing.

## INTRODUCTION

The role of bacteria as an anticancer agent has been recognized from very early studies. The German physicians W. Busch and F. Fehleisen separately observed that cancers regressed following accidental erysipelas (*Streptococcus pyogenes*) infections in patients [1]. Later William B. Coley found that some cancer patients who developed post-operative infection of bacteria were cured from their tumors [2]. *Salmonella typhimurium*, a facultive anaerobe which can grow in both necrotic and live tumor tissue, is one of the most broadly studied tumor-targeting bacteria. S*almonella* can colonize primary tumors as well as accumulate within metastases, rendering *S. typhimurium* an ideal anticancer agent [3–5].

VNP20009, a strain of *Salmonella typhimurium*, is genetically attenuated by deleting both *purI* and *msbB* and has been widely investigated [6, 7]. The tumor-targeting and efficacy of VNP20009 has been demonstrated in a variety of animal models [3, 8, 9] and has shown to be safe in a Phase I clinical trial [10]. VNP20009 has also been used as a vector to deliver potentially therapeutic genes such as pro-drug converting enzymes, cytokines and other genes [11, 12].

*S. typhimurium* A1 obtained after nitrosoguanidine (NTG) mutagenesis to induce auxotrophic mutations for leu and arg, selectively grew in tumor xenografts with rapid clearance in normal tissues [13]. *S. typhimurium* A1 is able to receive sufficient nutritional support from tumors. *S. typhimurium* A1-R was further isolated from A1 infection of a human colon tumor in nude mice. Compared to *S. typhimurium* A1, *S. typhimurium* A1-R has increased tumor-targeting capacity and antitumor efficacy against major types of cancers [4, 14–16]. Mechanistically, it was previously reported that tumor-targeting *Salmonella* could suppress tumor growth by inducing cell apoptosis, tumor necrosis as well as suppressing tumor angiogenesis [17, 18].

Autophagy (macroautophagy) is an important cellular response that targets long-lived proteins, damaged organelles and pathogens for lysosome-mediated degradation [19, 20]. Previous studies showed that *Salmonella typhimurium*, an intracellular pathogen, could be targeted by autophagy after infecting mammalian cells such as macrophages and epithelial cells [21, 22]. Autophagy targeting of *S. typhimurium* appears to protect host cells, which has been shown to inhibit the replication of the bacteria in infected cells [21, 22]. Moreover, autophagy inhibitors such as wortmannin promote the release of *S. typhimurium* from vacuoles, and allow them to grow rapidly in the cytosol of cells [23]. More permissive intracellular growth of *S. typhimurium* in autophagy-deficient MEF-Atg5(−/−) cells than wild-type fibroblasts MEF-Atg5(+/+) also demonstrated that autophagy plays a role in restricting the replication of *S. typhimurium* in the cytosol [24].

Based on these findings, we hypothesized that tumor-targeting *Salmonella* such as A1-R or VNP20009 may induce autophagy in human cancer cells, and inactivation of autophagy pathway may significantly enhance *Salmonella* tumor-targeting. In this study, we verified this hypothesis, highlighting an innovative combination therapy of autophagy blockage with tumor-targeting *Salmonella*.

## RESULTS AND DISCUSSION

### Tumor-targeting *Salmonella* A1-R and VNP20009 induce autophagy in human cancer cells

**Figure 1:**
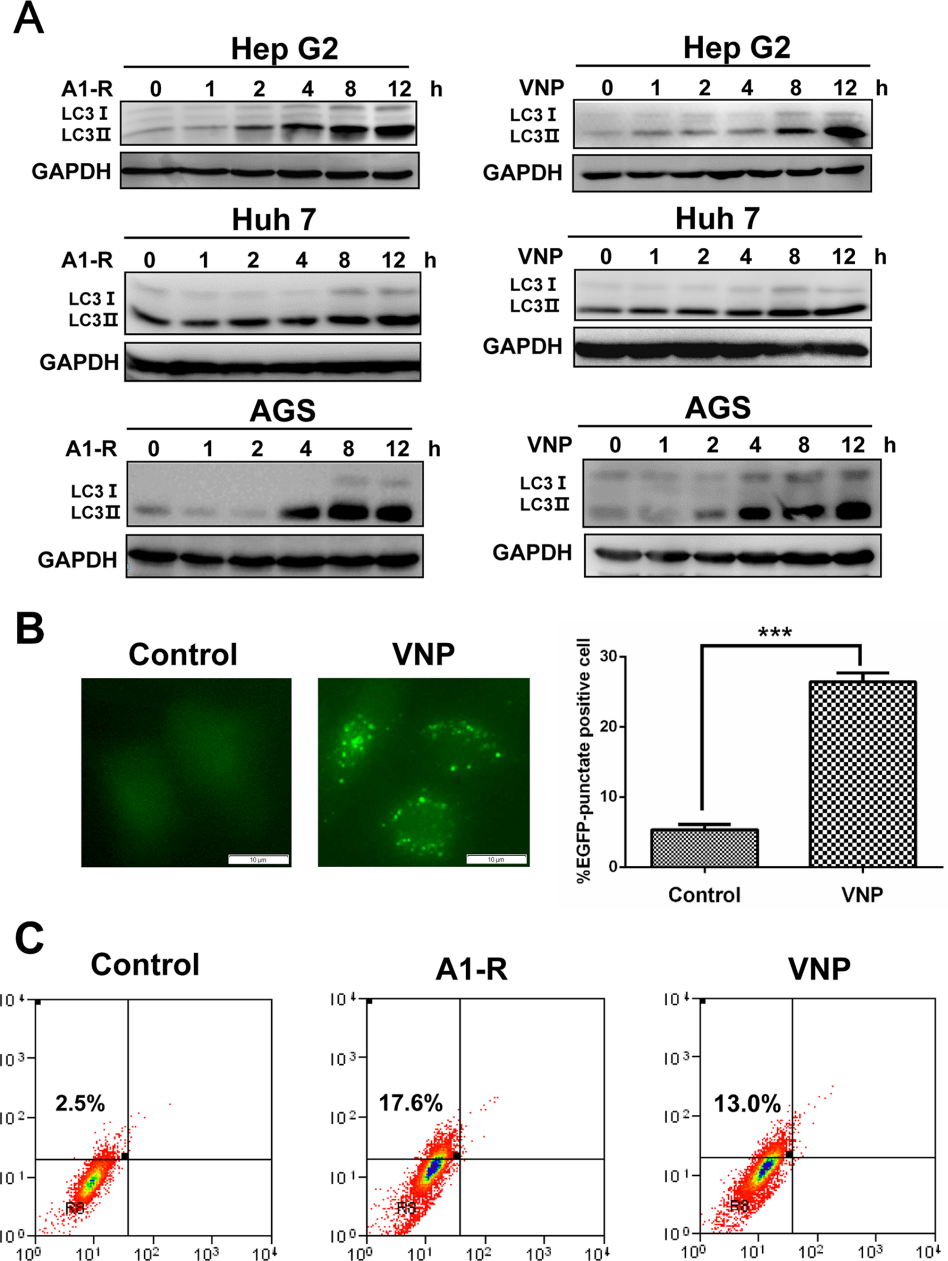
Tumor-targeting *Salmonella* A1-R and VNP20009 induce autophagy in human cancer cells (**A**) Autophagy response was measured by the conversion of LC3-I to lipidated LC3-II. Cancer cells (including Hep G2, Huh 7 and AGS) were harvested 0, 1, 2, 4, 8, and 12 hours (h) after infection of *S. typhimurium* (A1-R) or VNP20009 (VNP) and subjected to IB analysis. (**B**) Autophagy response measured by appearance of punctate vesicle structures. Hep G2 cells, stably expressing EGFP-LC3, were infected with VNP for 8 hours (hrs) and photographed under fluorescence microscopy. Cells with punctate vesicle structures, visualized by EGFP-LC3, were counted as autophagy-positive cells. Scale bars indicate length of 10 μm. (**C**) Autophagy measured by AO staining. Cells (4 × 10^5^) were seeded into 60 mm dishes and infected with or without *Salmonella* A1-R or VNP20009 and then stained with AO. FACS analysis was carried out 8 h post-infection as described in the Materials and Methods.

To determine whether tumor-targeting *Salmonella* A1-R and VNP20009 induce autophagy in cancer cells, we first determined the effect of *S. typhimurium* A1-R or VNP20009 infection on the formation of autophagosome membranes in cancer cells by detection of the conversion of LC3 I (microtubule-associated protein 1 light chain 3) to lipidated LC3 II, a classical marker of autophagy induced at the early stage and degraded at the late stage of autophagy [24, 25]. As shown in Figure 1A, *S. typhimurium* A1-R or VNP20009 infection induced conversion of LC3 I to LC3 II in HepG2 and Huh7 human liver cancer cells and AGS human gastric carcinoma cells.

Then we determined whether bacterial infection induces formation of puncta, another marker of autophagy [25, 26], in EGFP-LC3-expressing HepG2 cells (HepG2-EGFP-LC3). We found that VNP20009 induced a classical puncta formation in infected cells compared with control cells (Figure 1B). Furthermore, in the acridine-orange staining assay and FACS analysis for autophagy detection, we demonstrated that 17.6% and 13.0% of HepG2 cells infected with A1-R and VNP20009, respectively, had the accumulation of acidic vesicular organelles (AVO, a marker of autophagy), while only 2.5% of uninfected cells had AVOs (Figure 1C). These results demonstrated that tumor-targeting *Salmonella* A1-R and VNP20009 induced autophagy in human cancer cells.

### Autophagy restricts the growth of tumor-targeting *Salmonella* in cancer cells

**Figure 2:**
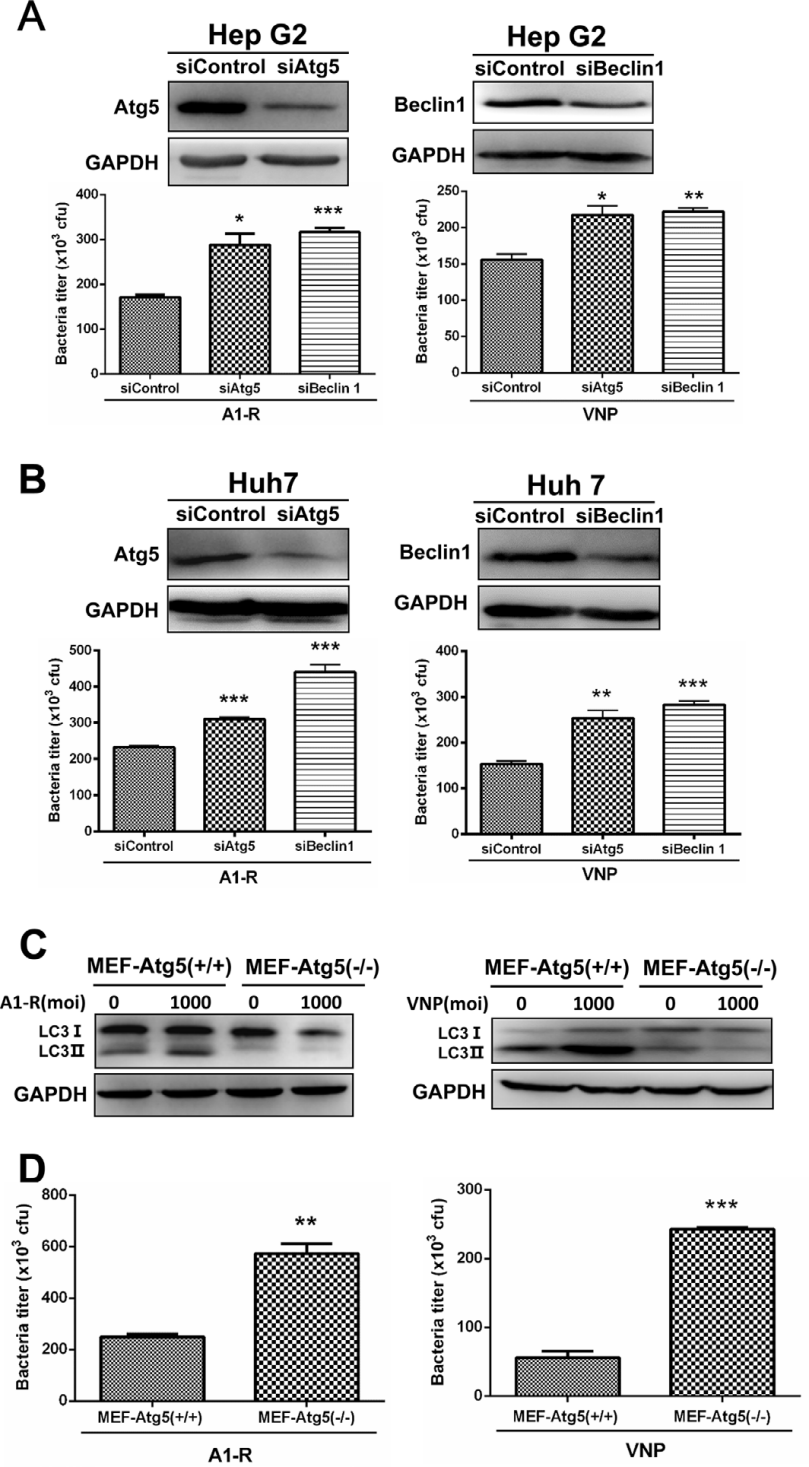
Autophagy restricts the growth of tumor-targeting *Salmonella* in cancer cells (**A**–**B**) Autophagy restricts the replication of *S. typhimurium* (A1-R) or VNP20009 (VNP) in human liver-cancer cells. Hep G2 (A) and Huh 7 (B) cells (7 × 10^4^) that had been transfected with siATG5 or siBcelin 1 for 48 hrs, were plated in 24-well culture plates at 70% confluence 16–24 h before bacterial infection. The cells were infected with tumor-targeting *Salmonella* A1-R or VNP at a MOI of 1000. After 8 hrs of infection, the cells were washed with PBS and lysed in buffer (PBS with 0.1% TritonX-100) and plated on agar. After incubation overnight at 37°C, CFUs were counted to estimate the intracellular titer of A1-R or VNP. At least three replicates were plated in this experiment. The knockdown of Atg5 and Beclin 1 was monitored by IB analysis. Liver-cancer cells Hep G2 and Huh 7 transfected with siControl, siATG5 or siBcelin 1 for 96 hrs were subjected to IB analysis for expression of the indicated proteins. (**C**) A1-R or VNP infection induced autophagy in Wild-Type (Atg5+/+) MEFs, but not in autophagy-deficient (Atg5−/−) MEFs. Cells were harvested 2 h after A1-R or VNP's infection and subjected to IB analysis to detect the conversion of LC3-I to lipidated LC3-II. (**D**) Wild-Type (Atg5+/+) MEF and KO (Atg5−/−) MEFs cells (7 × 10^4^) were plated in 24-well culture plates 16–24 h before bacterial infection. The intracellular bacterial measurements then conducted as described above. The results are presented as mean value ± SE from three independent experiments with each running in triplicate. **p < 0.01, ***p < 0.001.

To determine the biological role of tumor-targeting *Salmonella*-induced autophagy, we blocked the autophagy pathway via siRNA, silencing the autophagy-essential genes Atg5 or Beclin1 and determined the effect on the intracellular bacterial multiplication. As shown in Figure 2A and Figure 2B, suppression of autophagy via Atg5 and Beclin1 knockdown significantly enhanced bacterial replication in both HepG2 (Figure 2A) and Huh 7 (Figure 2B) cells. To confirm this, we further utilized a MEF cell line with a knock-out of Atg5 locus (Atg5^−/−^) which leads to deficiency in autophagy, since Atg5 is essential for the early steps in autophagosome formation [27]. By determining the conversion of LC3-I to LC3-II with Western blot analysis, we firstly confirmed that *S. typhimurium* A1-R or VNP20009 induced autophagy in MEF-Atg5(+/+) (MEF cells expressing wild type Atg5) but not MEF-Atg5(−/−) (Figure 2C). Then, we investigated the role of autophagy in the growth of *S. typhimurium* A1-R and VNP20009 by infecting the pair of MEF cell lines. As shown in Figure 2D, autophagy-deficient MEF-Atg5(−/−) showed greater intracellular *S. typhimurium* A1-R and VNP20009 proliferation when compared with wide-type MEF-Atg5(+/+). Collectively, these results demonstrate that the autophagic response induced by tumor-targeting *Salmonella* plays a protective role for infected cancer cells.

### Blockage of autophagy pathway enhances *Salmonella*-mediated anticancer therapy

**Figure 3:**
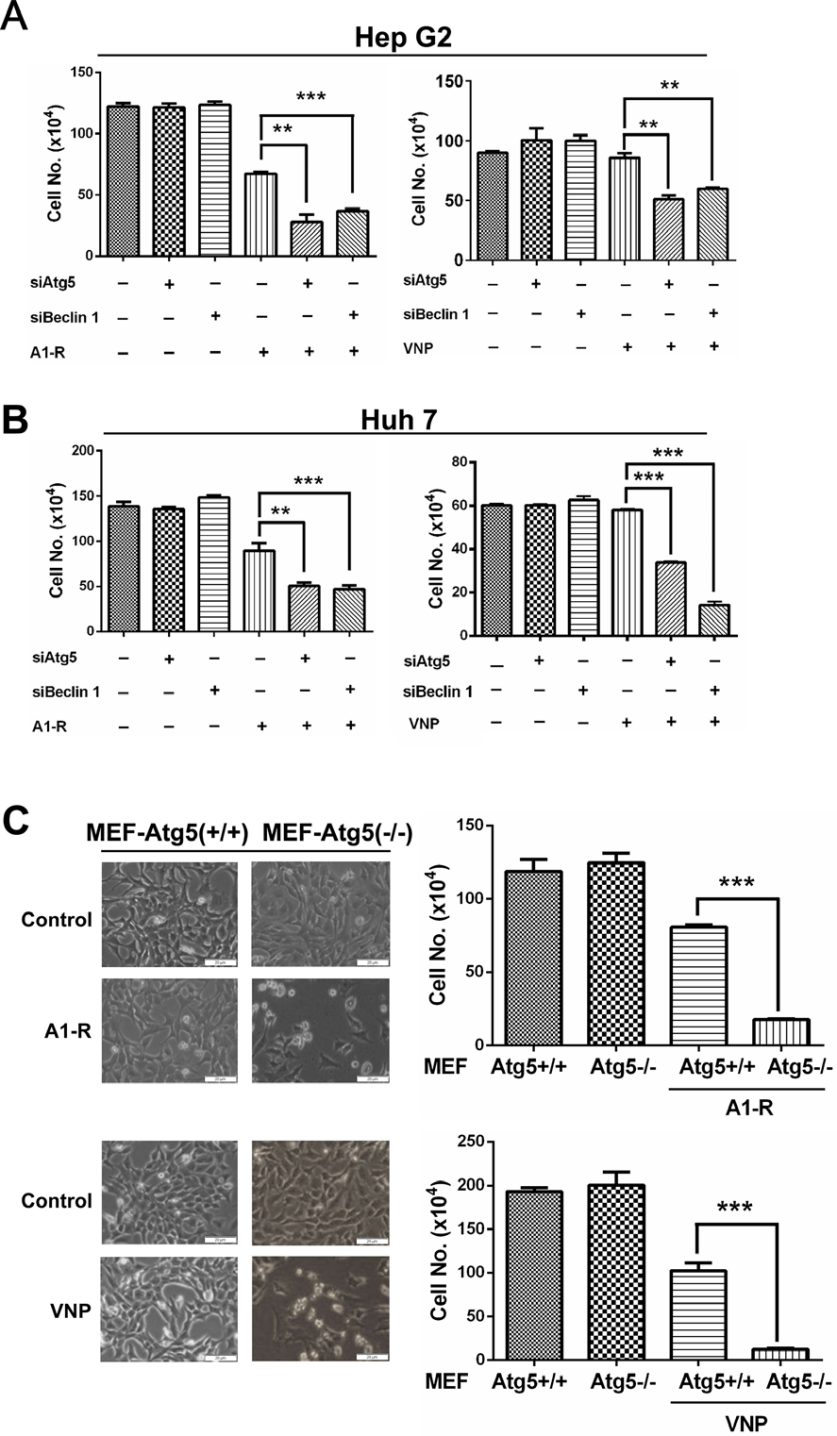
Blockage of autophagy pathway enhances *Salmonella*-mediated anticancer therapy (**A**–**B**) Liver cancer cells Hep G2 (A) and Huh 7 (B) cells (3 × 10^4^), that had been transfected with siAtg5, siBcelin 1 or siControl for 48 hrs, were plated in 12-well plates 16–24 h before A1-R or VNP infection. Infection was carried out as described in the Materials and Methods at a moi of 1000. (**C**) MEF-Atg5(+/+) or MEF-Atg5(−/−) cells (4 × 10^4^) were plated in 60-mm dishes 16–24 h before bacterial infection. Then, bacterial infection, morphologic observation, and cell counting measurements were conducted as described in the Materials and Methods. All data presented are representative results of at least two independent experiments. Scale bars indicate length of 20 μm. All cell counting results are presented as mean value ± SE from three independent experiments, with each running in triplicate. **p < 0.01, ***p < 0.001.

Having established that autophagy restricts the growth of tumor-targeting *Salmonella* in cancer cells, we next investigated a hypothesis that blockage of autophagy pathway enhances the anticancer efficacy of *S. typhimurium* A1-R or VNP20009 and further retards cancer cell growth. As shown in Figure 3A and 3B, blocking the autophagy pathway via knocking down of Atg5 or Beclin1 significantly enhanced bacterial inhibition of proliferation in both HepG2 and Huh7 cells, while siRNA silencing of Atg5 or Beclin1 alone did not notably affect cell proliferation. Moreover, the enhanced cytotoxic effect of autophagy inhibition combined with bacterial treatment was further confirmed by using infected MEF-Atg5(+/+) (autophagy-competent) versus MEF-Atg5(−/−) (autophagy-deficient) cells. As shown in Figure 3C, morphologic observation and cell counting showed that MEFs-Atg5(−/−) showed greater cell death compared with wide-type MEFs-Atg5(+/+) cells upon *Salmonella* infection. These findings demonstrate that blockage of the autophagic pathway significantly enhances *Salmonella* tumor-targeting.

### Blockage of autophagy pathway enhances *Salmonella*-induced apoptosis

**Figure 4:**
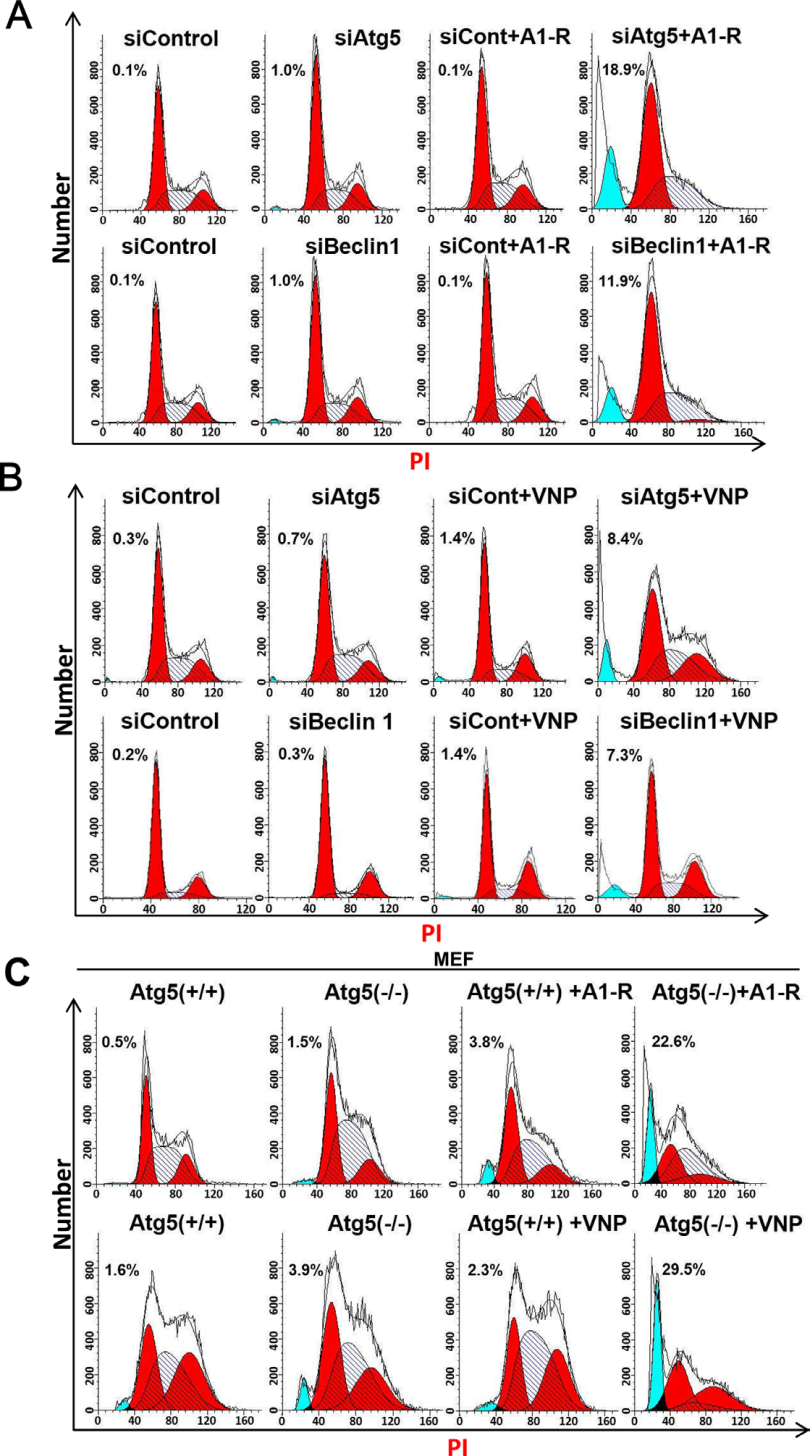
Blockage of autophagy pathway enhances *Salmonella*-mediated apoptosis (**A**) Blockage of the autophagy pathway enhanced cell apoptosis upon *S. typhimurium* (A1-R) infection. Hep G2 cells (4 × 10^5^ cells) that had been transfected with siATG5, or siBeclin 1, or siControl for 48 hrs were seeded in 60-mm dishes. Bacterial infection was conducted, and cells were subjected to PI staining and FACS analysis 36 h post-infection. The percentage of cells in the sub-G 1 phase is indicated. (**B**) Blockage of autophagy enhanced cell apoptosis upon VNP20009 (VNP) infection. Hep G2 cells (2 × 10^5^) that had been transfected with siATG5, or siBeclin 1, or siControl for 48 hrs were seeded into 60-mm dishes. Cells were infected with VNP and subjected to PI staining and FACS analysis. The percentage of cells at the sub-G 1 phase is indicated in the figure. (**C**) Autophagy-deficient MEF-Atg5(−/−) cells exhibited increased apoptosis compared with autophagy-competent MEF-Atg5(+/+) cells upon A1-R or VNP infection. MEF-Atg5(−/+), cells at 4 × 10^4^ (A1-R) and 3 × 10^4^ (VNP) cells were seeded in 60-mm dishes 16–24 h before bacterial infection. Bacterial infection, PI staining, and FACS analysis were conducted as described above. These data were representative results of at least two independent experiments.

Having established that blockage of the autophagy pathway combined with *S. typhimurium* A1-R or VNP20009 infection leads to higher intracellular bacteria colonization (Figure 2) and enhanced cell killing (Figure 3), we then investigated how autophagy inhibition sensitizes cells to bacterial infection. PI staining and FACS analysis revealed that the blockage of autophagy via Atg5 or Beclin1 siRNA silencing significantly enhanced *S. typhimurium* A1-R and VNP20009-induced apoptosis, as demonstrated by the appearance of a sub-G1 peak in FACS analysis (Figure 4A and 4B). To confirm the effect of the combination therapy on apoptotic induction, *S. typhimurium* A1-R or VNP20009 infected MEFs-Atg5(+/+) and MEFs-Atg5(−/−) were subjected to apoptosis analysis by PI staining and FACS analysis. Similarly, we found that *S. typhimurium* A1-R infection of MEFs-Atg5(−/−) induced 22.6% apoptotic cells, but only 3.8% in infected MEFs-Atg5(+/+), while VNP20009 infection led to 29.5% MEFs-Atg5(−/−) cells undergoing apoptosis, but only 2.3% in infected MEFs-Atg5(+/+) (Figure 4C). These findings demonstrated that blockage of the autophagy pathway significantly enhanced tumor-targeting *Salmonella*-induced apoptosis.

### Pharmaceutical inhibition of autophagy pathway enhances the anticancer efficacy of tumor-targeting *Salmonella*

**Figure 5:**
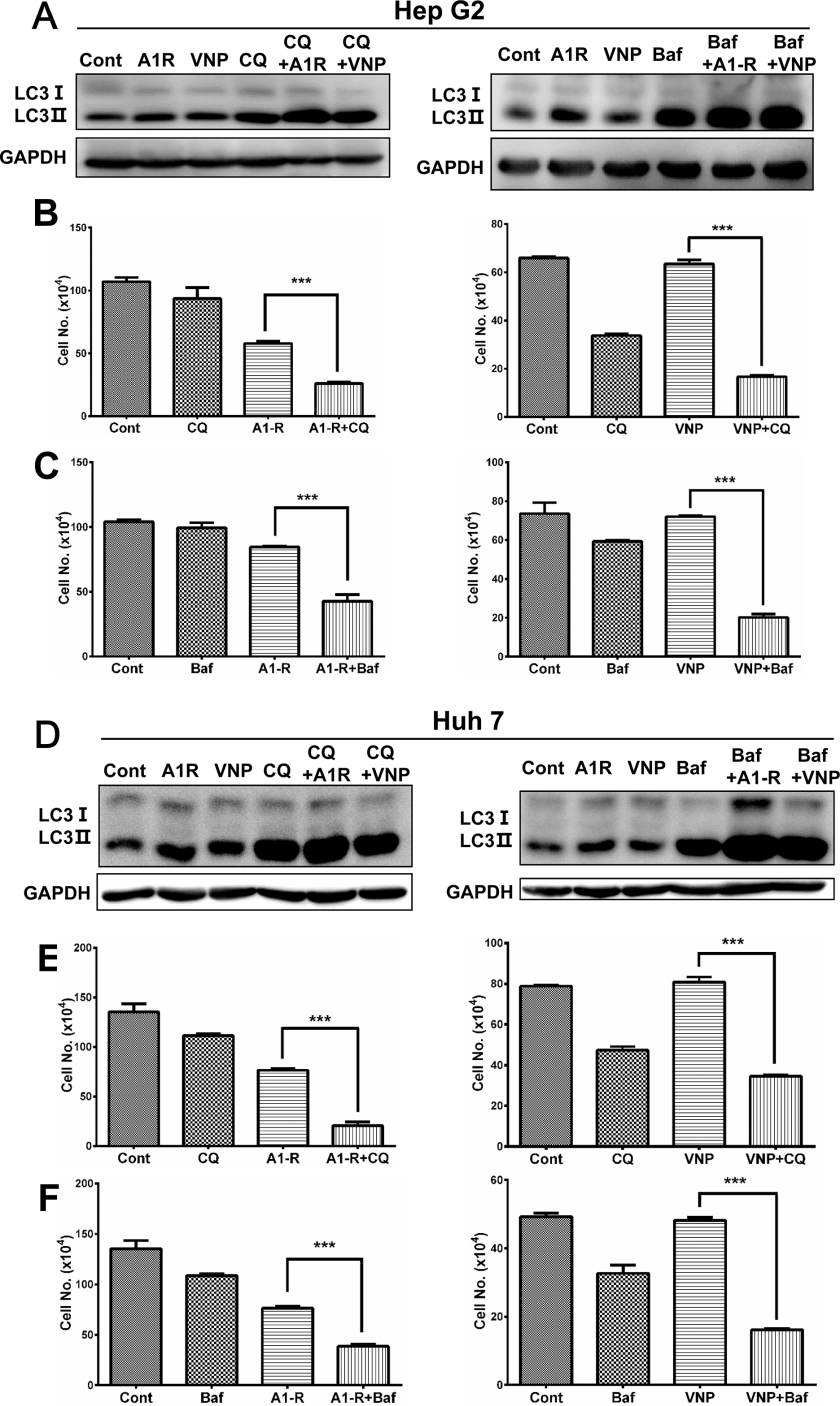
Pharmaceutical inhibition of autophagy pathway enhances the anticancer efficacy of tumor-targeting *Samonella* (**A** and **D**) Autophagic-flux analysis after treatment with CQ and Baf A1. Hep G2 (A) or Huh 7 (D) cells were infected with *S. typimurium* (A1-R) or VNP20009 (VNP) for 4 hrs or not infected. Cells were then incubated with or without CQ (5 μM) or Baf A1 (2 nM) for 6 hrs. Cells were then subjected to IB analysis with GAPDH as a loading control. (**B** and **E**) Autophagy inhibition by CQ enhanced A1-R- or VNP-mediated cell killing of Hep G2 (B) and Huh 7 cells (E). Bacterial infection and 5 μM CQ treatment were performed as in A. Cell counting was conducted at the indicated time points. (**C** and **F**) Autophagy inhibition by Baf A1 enhanced A1-R- or VNP20009-mediated cell killing in Hep G2 (C) and Huh 7 cells (F). A1-R infection and 2 nM Baf A1 treatment was performed as in D. Cells infected with VNP for 4 h or uninfected and treated with or without 1 nM Baf A1. Cell counting was conducted at the indicated time points. All results are representative of at least two independent experiments. All cell-counting results are presented as mean value ± SE from three independent experiments, each running in triplicate. ***p < 0.001.

Since genetic inhibition of the autophagy pathway via siRNA silencing significantly enhanced cytotoxicity of tumor-targeting *Salmonella*, we further hypothesized that pharmacological inhibition of autophagy would enhance bacteria-induced cell-growth suppression. For this purpose, we used two classical autophagy inhibitors CQ and BafA1 which prevent the late stages of autophagic-flux by inhibiting the fusion of autophagosomes with lysosomes and subsequent lysosomal protein degradation [24, 26]. We performed autophagic-flux analysis by treating bacterial infected cells with CQ and BafA1 respectively, then found the accumulation of LC3 II was remarkably enhanced compared with either drug-treated or bacterially-infected alone, indicating that autophagic flux induced by *Salminella* was intact (Figure 5A). As a result, autophagy inhibition with either CQ or BafA1 significantly enhanced *Salmonella*-induced cell growth suppression compared with bacterial infection alone (Figure 5B and 5C). Similarly, both CQ and BafA1 treatment greatly enhanced the cytotoxicity of *S. typhimurium* A1-R and VNP20009 in Huh7 cells (Figure 5D–5F). These findings indicate that pharmacological inhibition of autophagy significantly enhances *Salminella* tumor-targeting.

In summary, we report for the first time that tumor-targeting *Salmonella* induces autophagy in human cancer cells. The autophagy response of cancer cells upon bacterial infection serves as a cellular defense signal. More importantly, genetic or pharmaceutical inhibition of the autophagy pathway significantly enhances intracellular replication of tumor-targeting *Salmonella* and cytotoxicity. These findings revealed a novel interaction mechanism between cancer cells and tumor-targeting *Salmonella* from the perspective of autophagy and provide proof-of-concept evidence for the combination therapy of autophagy inhibitors with tumor-targeting *Salmonella*, further demonstrating its future clinical potential [31].

## MATERIALS AND METHODS

### Cell lines, culture, and reagents

Human liver-cancer cell lines Huh7, HepG2, human gastric adenocarcinoma cell line (AGS) and MEF-Atg5(−/−) and MEF-Atg5(+/+) cells were obtained from the American Type Culture Collection, and cultured in Dulbecco's Modified Eagle's Medium (DMEM, Hyclone Labs, Logan, UT), containing 10% fetal bovine serum (FBS) and 1% penicillin-streptomycin solution, at 37°C with 5% CO_2_. Chloroquine (CQ) and bafilomycin A1 (Baf A1) were purchased from Sigma (St. Louis, Mo).

### Establishment of HepG2-EGFP-LC3 stable cell lines

HepG2 cells stably expressing an EGFP-LC3 fusion protein were established as described [28]. Briefly, cells were seeded in 6-well plates and transfected with 3 μg pEGFP-LC3 plasmid using Lipofectamine 2000 (Invitrogen). Cells with enhanced EGFP fluorescence were selected with an MoFlo^™^ XDP Cell Sorter (Beckman Coulter) and cultured in complete cell culture medium containing G418 at 200 μg/mL. The autophagy induced by A1-R or VNP20009 infection was measured by the appearance of punctate vesicle structure and photographed under fluorescence microscopy (Leica, Wetzlar, Germany).

### *Salmonella* strains and bacterial infection

Tumor-targeting *Salmonella typhimurium* A1-R (AntiCancer, Inc., San Diego, CA) and Lipid A-modified (*msbB* ^−^) and auxotrophic (*purI* ^−^) *Salmonella typhimurium* VNP20009 (ATCC, USA) were cultured in modified Luria–Bertani (LB) medium to late-log phase as previously described [4, 6]. Cancer cells were cultured in DMEM with 10% FBS and antibiotics (pencillin and streptomycin), for approximately 16–24 hours (h) before bacterial infection. Medium was changed to antibiotic-free medium 2 h before infection. The bacteria were diluted in DMEM and added to the cancer cells in a ratio of 1000 MOI, and placed in an incubator at 37°C. After 2 h, the cells were rinsed three times with DMEM and then cultured in medium containing 10% FBS with gentamicin sulfate (150 μg/mL) to kill external bacteria. After incubating 2 h, the cells were washed three times with DMEM, and then cultured in medium containing 10% FBS. For siRNA experiments, cells were transfected with the indicated siRNA and infection was performed at indicated hours after gene knockdown. (please see below).

### Colony-forming unit (cfu) assay

Cells were plated in 24-well plates at 7 × 10^4^ cells/well and cultured to approximately 70% confluence 16–24 h before bacterial infection. In order to determine the number of intracellular bacteria, these cells were washed by PBS twice, digested and lysed by 0.25% trypsin and 0.1% TritonX-100, respectively. Then, the diluted lysates were plated on agar. After incubated overnight at 37°C, CFUs were counted to estimate the intracellular titer of A1-R or VNP20009. This assay was repeated at least three times.

### siRNA silencing

Liver cancer cells HepG2 or Huh7 cells were transfected with siRNA oligonucleotides using the Lipofectamine 2000 reagent according to the manufacturer's instructions. The sequence of siRNAs are as follows: for Atg5 [29], siAtg5: 5′-GGATGAGATAACTGAAAGG-3′; for Beclin1 [30], siBeclin1: 5′-CAGUUUGGCACAAUCAAUA-3′; for control scrambled siRNA, siControl: 5′-UUCUCCGAAC GUGUCACGUTT-3′. The siRNAs were purchased from GenePharma (Shanghai, China).

### Immunoblotting analysis

Cell lysates were prepared for immunoblotting (IB) analysis, using antibodies against ATG5, Beclin1 (Cell Signaling Inc, USA), LC3 and GAPDH (Sigma).

### Propidium iodide (PI) staining

Cells were harvested and fixed in 70% ethanol at −20°C overnight, then stained with propidium iodide (36 μg/mL, Sigma) containing RNase (10 mg/mL; Sigma) at 37°C for 15 minutes, and analyzed by flow cytometry (CyAn^™^ ADP, Beckman Coulter) for cell-cycle profiling and apoptosis. Apoptosis was measured by the percentage of cells in the sub-G1 population. Data were analyzed with ModFit LT software.

### Acridine orange (AO) staining for autophagy detection

Acridine orange (Sigma,) staining was performed according to a published protocol [24, 26]. Briefly, cells were stained with 1 mM acridine orange in PBS containing 5% FBS at 37°C for 15 minutes. Cells were washed and observed under fluorescence microscopy (BX-51, Olympus Corp., Tokyo, Japan).

### Cell counting

Cells that were transfected with the indicated siRNAs were split 48 h post transfection and seeded into 12-well plates with 3 × 10^4^ cells/well (VNP20009) or 4 × 10^5^ cells/60 mm dish *S. typimurium* (A1-R). For cell counting at indicated time points, cell culture medium was removed and PBS was used to wash cells three times. The cells were then treated with 0.25% trypsin and resuspended with DMEM containing 10% FBS. The cell number was then counted. This assay was repeated at least three times.

### Statistical analysis

All data are presented as mean ± SEM. The unpaired 2-tailed *t* test was used for the comparison of parameters between two groups and the statistical significance of differences between groups was assessed using GraphPad Prism5 software. Three levels of significance (*P < 0.05, **P < 0.01, ***P < 0.001) were used.
